# A method to decompose spectral changes in *Synechocystis* PCC 6803 during light-induced state transitions

**DOI:** 10.1007/s11120-016-0248-8

**Published:** 2016-03-25

**Authors:** Alonso M. Acuña, Radek Kaňa, Michal Gwizdala, Joris J. Snellenburg, Pascal van Alphen, Bart van Oort, Diana Kirilovsky, Rienk van Grondelle, Ivo H. M. van Stokkum

**Affiliations:** 1 Faculty of Sciences, Institute for Lasers, Life and Biophotonics, Vrije Universiteit Amsterdam, De Boelelaan 1081, 1081 HV Amsterdam, The Netherlands; 2 Laboratory of Photosynthesis, Centre Algatech, Institute of Microbiology, Opatovický Mlýn, 379 81 Třeboň, Czech Republic; 3 Institute for Integrative Biology of the Cell (I2BC), CEA, CNRS, Univ Paris-Sud, Université Paris-Saclay, 91198 Gif-sur-Yvette Cedex, France; 4 Swammerdam Institute for Life Sciences, University of Amsterdam, 1098 XH Amsterdam, The Netherlands

**Keywords:** Cyanobacteria, Spectrally resolved fluorometry, Singular value decomposition, Time-resolved spectroscopy

## Abstract

Cyanobacteria have developed responses to maintain the balance between the energy absorbed and the energy used in different pigment-protein complexes. One of the relatively rapid (a few minutes) responses is activated when the cells are exposed to high light intensities. This mechanism thermally dissipates excitation energy at the level of the phycobilisome (PB) antenna before it reaches the reaction center. When exposed to low intensities of light that modify the redox state of the plastoquinone pool, the so-called state transitions redistribute energy between photosystem I and II. Experimental techniques to investigate the underlying mechanisms of these responses, such as pulse-amplitude modulated fluorometry, are based on spectrally integrated signals. Previously, a spectrally resolved fluorometry method has been introduced to preserve spectral information. The analysis method introduced in this work allows to interpret SRF data in terms of species-associated spectra of open/closed reaction centers (RCs), (un)quenched PB and state 1 versus state 2. Thus, spectral differences in the time-dependent fluorescence signature of photosynthetic organisms under varying light conditions can be traced and assigned to functional emitting species leading to a number of interpretations of their molecular origins. In particular, we present evidence that state 1 and state 2 correspond to different states of the PB-PSII-PSI megacomplex.

**Electronic supplementary material:**

The online version of this article (doi:10.1007/s11120-016-0248-8) contains supplementary material, which is available to authorized users.

## Introduction

Excess light is a hazard all photosynthetic organisms have to deal with (Demmig-Adams et al. 2014). Long-lived excited chlorophyll species lead to the formation of reactive oxygen species (ROS) that damage the photosystems and impair growth or even lead to death (Nishiyama et al. 2001). High-energy-dependent as well as light-adaptive mechanisms have evolved to cope with varying excitation energy fluxes (Papageorgiou 1996; Papageorgiou et al. 2007; Joshua and Mullineaux 2004; Mullineaux and Allen 1990, 1986). Cyanobacterium *Synechocystis* PCC 6803 (hereafter, *Synechocystis*) possesses a phycobilisome (PB) antenna that harvests mainly orange light which is hardly absorbed by photosystem (PS) I or PSII (Shevela et al. 2013). Also, a megacomplex of PB, PSII, and PSI has been described (Liu et al. 2013; Steinbach et al. 2015) in which the energy absorbed by PB can be transferred to PSII or PSI. In vivo, the abundance and the dynamics of excitation energy transfer in this megacomplex are still debated (Chukhutsina et al. 2015).

Non-photochemical fluorescence quenching (NPQ) at the level of the PB antenna has been observed when high photon fluxes photoconvert the orange carotenoid protein (OCP) to its red form (OCP^r^) (Wilson et al. 2008) which then binds to the phycobilisome thereby inducing fluorescence quenching (Gwizdala et al. 2011). Cyanobacteria also regulate the supply of electronic excitations to PSI and PSII by means of state transitions (Kirilovsky et al. 2014; Mullineaux and Emlyn-Jones 2005; Vernotte et al. 1990; Kaňa et al. 2012). In state 1, the PB antenna transfers energy to PSII predominantly. As the electron transport chain from PSII to PSI gets reduced, a rearrangement of the building blocks in and around the thylakoid membrane allows the antenna to supply PSI with more and PSII with less energy. This is the state 1 to state 2 transition. In darkness, *Synechocystis* cells have been reported to be in state 2 due to the respiratory activity (Campbell et al. 1998; Liu 2015; Mullineaux 2014). Changes in fluorescence allow us to follow the activation (as well as deactivation or persistence) of the mechanisms described above.

The spectral properties of the different subunits of the cyanobacterial photosynthetic apparatus have been studied in the past (see e.g., Komura and Itoh (2009) and references therein). The presence of phycocyanin (PC) and allophycocyanin (APC) in the PB antenna leads to emission in the 655 and 670 nm regions (Glazer and Bryant 1975; Cho and Govindjee 1970; Gwizdala et al. 2011). After excitation of the PB with 590 nm light, energy transfer to the photosystems I and II results in Chl a emission around 680–690 nm (Tian et al. 2011). The spectral evolution of the fluorescence on the ps and ns time scales thus results in a steady-state spectrum characteristic, for e.g., the PB-PSII complex.

For a systematic study, given the multiplicity and a variety of mechanisms cyanobacteria possess to control the photosynthetic electron transport (Kirilovsky et al. 2014; Liu 2015; Govindjee and Shevela 2011), we have used model systems: (i) an in vitro experiment, where the OCP-induced energy dissipating mechanism was reconstituted and (ii) two mutants of *Synechocystis*, lacking either PSI (ΔPSI) or PSII (ΔPSII). Such model systems are expected to generate simplified signals in comparison to the complete in vivo system and allow us to validate the new method of analysis. They provide important contributions to understanding the bigger picture, i.e., that of the wild-type organism.

These processes can be investigated using spectrally resolved fluorometry (SRF) using a set-up with multiple independently controllable excitation sources, e.g., LEDs with various spectral profiles (Lambrev et al. 2010). In this configuration, one LED can be used to acquire full fluorescence spectra using a specific light intensity and spectral profile, whereas a second LED can be used to induce changes to the photosynthetic apparatus using a different intensity or different spectral characteristics. The result is a data matrix that displays levels of fluorescence emission as a function of wavelength and time (Kaňa et al. 2012; Kaňa et al. 2009; Lambrev et al. 2010). In this work, the data matrices are then analyzed with a model-based approach, assuming that at any timepoint, the spectra can be described as a linear combination of species-associated spectra (SAS). The SAS are the same for all time points, but their relative contributions vary with time. In contrast to PAM signals, where all fluorescence above 700 nm is integrated, the time-resolved spectrum can be analyzed to resolve the SAS and the time-dependent contributions of the different species, leading to a number of interpretations of their molecular origins.

## Materials and methods

### Isolated PB and OCP to form quenching complexes

Isolation of PB and OCP is described in detail in Gwizdala et al. (2011). PBs were stored and examined in a 0.8 M potassium phosphate buffer. In this experiment, the molecular ratio between the PBs and OCP was 1:37 (±1).

### Cell cultures

For experiments with closing of PSII RCs in vivo (as described in the “Closing of reaction centers during fluorescence induction by saturating light in vivo” section), we used wild-type *Synechocystis* PCC 6803 (a glucose-tolerant derivative) kindly provided by Devaki Bhaya (Department of Plant Biology, Carnegie Institution for Science, Stanford, California, USA); it was cultivated in a modified BG-11 medium (Stanier et al. 1971) in a photobioreactor [model FMT 150.2/400, Photon Systems Instruments; for details, see Nedbal et al. (2008)] as previously described by van Alphen and Hellingwerf (2015). BG-11 was supplemented with 10 mM NaHCO_3_. A mixture of CO_2_ in N_2_ (150 mL min^−1^) was used to provide a constant supply of CO_2_; the pH was set to 8.0 by automatically adjusting the pCO_2_ using a gas mixing system (GMS150, Photon Systems Instruments). The photobioreactor was run as a turbidostat, which allowed continuous growth at a set optical density (OD) at 730 nm of 0.4 ± 2 % (OD_730_ = 1 ≈ 10^8^ cells mL^−1^), as measured by a benchtop photospectrometer (Lightwave II, Biochrom). Seventy-five µmol of photonsˑm^−2^ s^−1^ of orange-red light (λ_max_ 636 nm, 20 nm full-width at half-maximum) was provided to the cells, using an integrated LED panel, which yielded a doubling time of approximately 9 h. The temperature was set to 30 °C and maintained to within 0.2 °C. For other experiments, described in the “In vivo fluorescence induction with orange light in wild-type and PSI-, and PSII-deficient mutants of *Synechocystis*” section, we used freshwater cyanobacteria *Synechocystis* PCC 6803 (wild-type and its mutants); they were cultivated in BG 11 medium, in an orbital shaking incubator, at 28 °C and at a constant irradiance of 40 μmol of photons m^−2^ s^−1^ of PAR (photosynthetically active radiation, 400–700 nm). The specific mutants without PSI [ΔPSI, without PsaA and PsaB proteins; for details, see Shen et al. (1993)] or without PSII [ΔPSII without CP47 and CP43 proteins and with at most 10 % of PSII-RC; for details, see Komenda et al. (2004)] were used for our measurements.

### Time-resolved fluorescence spectra at room temperature

Two set-ups were employed, one in Amsterdam and the other in Třeboň. The set-up in Třeboň has been described by Kaňa et al. (2009) and it was used for experiments presented in the “In vivo fluorescence induction with orange light in wild-type and PSI-, and PSII-deficient mutants of *Synechocystis*” section. The set-up that was used in Amsterdam to track PB fluorescence quenching in vitro (“OCP-related PB fluorescence quenching in vitro” section) and closing of RCs in vivo (“Closing of reaction centers during fluorescence induction by saturating light in vivo” section) is described here. This set-up has been originally developed for measurements on leaves. A detailed description can be found in Lambrev et al. (2010). A modified geometry allowed full fluorescence spectra to be acquired from samples in solution (Figure S1A). LEDs of several spectral characteristics can be easily interchanged as needed for a specific light protocol (Figure S1B). During the quenching experiments on isolated PB (“OCP-related PB fluorescence quenching in vitro” section), orange light (590 nm; 300 µmol of photonsˑm^−2^ s^−1^) was used as measuring light (ML), while for OCP pre-conversion (placed in a bath at 4 °C) to OCP^r^, high-intensity white light (5000 µmol of photonsˑm^−2^ s^−1^) was used (Gwizdala et al. 2011). The measurement protocol consists of, first, continuous exposition of the isolated PB to ML, then addition, in one step, of OCP^r^ to the PB. In the case of the in vivo measurement (“Closing of reaction centers during fluorescence induction by saturating light in vivo” section), cells were exposed to 1500 µmol of photonsˑm^−2^ s^−1^ of orange light during one second. In both the cases, the signals were collected (integration time) every 100 ms. In addition, a very low-intensity experiment was carried out with 1 µmol of photonsˑm^−2^ s^−1^ of orange light during one minute and integration time of 10 s. Fluorescence is collected via a lens that focuses them into a fiber connected to a CCD spectrometer (USB2000+, OceanOptics). Home-made protocols written in National Instruments LabView and Wolfram Mathematica were used to carry out the data acquisition and the data analysis, respectively.

### Protocol for spectrally resolved fluorescence induction measurements on intact living cells

Fluorescence experiments to monitor state transitions were performed by means of a spectrally resolved fluorescence induction (SRFI) method (Kaňa et al. 2009). In the “In vivo fluorescence induction with orange light in wild-type and PSI-, and PSII-deficient mutants of *Synechocystis*” section, fluorescence emitted by whole *Synechocystis* cells was sampled every 90 ms under continuous illumination for a period of 12 min. Experiments were made with control cells without inhibitors (*non*-*treated*, hereafter) and those treated with 3-(3′,4′-dichlorophenyl)-1,1-dimethylurea (DCMU), as described previously in Kaňa et al. (2012). Cells were dark adapted for 20 min before SRFI measurements to induce state 2. The maximal fluorescence [F_m_ or, if light-adapted, F′_m_; for details of nomenclature, see Krause and Weis (1991)] reflecting full closure of the PSII reaction centers was induced by a saturation light pulse (590 nm; Δt 200 ms; 1200 µmol of photonsˑm^−2^ s^−1^). The first and last pulses were applied during a period of darkness. The evolution of the spectrally resolved fluorescence signal, induced by orange actinic irradiation, was then measured during 10 min.

## Analysis of the data matrix

Acquired fluorescence spectra (*n* wavelengths) at *m* time points can be represented by an (*m* × *n*) data matrix Ψ. Below, we describe the procedure to extract species-associated spectra (SAS) and their time evolution.

The singular value decomposition (SVD) procedure (Golub and Van Loan 1996) decomposes Ψ according to

1 $$\varPsi = U \cdot S \cdot V^{T}$$

into an (*m* × *m*) matrix ***U***, where the *m* columns are called the *left singular vectors* (*lsv*); the (*m* × *n*) diagonal matrix ***S*** whose diagonal elements (*s*_*1*_ *, s*_*2*_ *, s*_*3*_ *…*) are called the singular values and the transpose of the (*n* × *n*) matrix ***V***, ***V*** ^**T**^, where the *n* rows are called the *right singular vectors* (*rsv*). In this study, the data matrix is constituted by spectra arrayed in time. Hence, the *rsv* which are significantly different from the noise are a linear combination of the SAS and the accompanying *lsv* are a linear combination of their time-dependent concentrations. We seek a mathematical transformation to resolve these SAS and their concentrations.

**Fig. 1:**
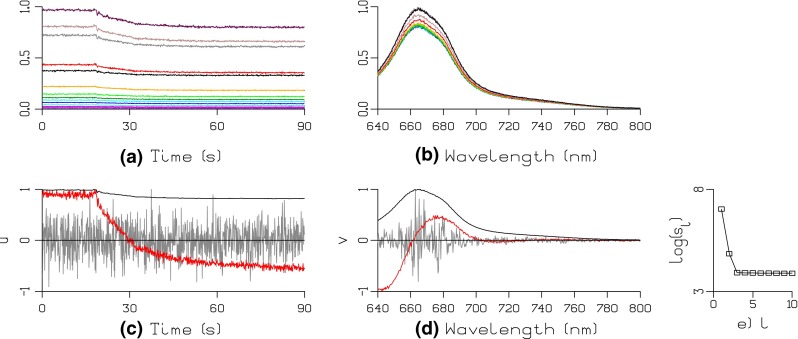
Visualization of the acquired dataset (**a**, **b**) and SVD (**c**, **d**, **e**) after a quenching experiment on isolated PB and pre-converted OCP^r^ (added at *t* = 18 s). The data matrix was obtained after continuous illumination of isolated PB in 0.8 M phosphate buffer with 590 nm light (300 µmol photons m^−2^ s^−1^). Fluorescence spectra were acquired every 100 ms. For selected timepoints, acquired spectra in the range 640–800 nm (**b**) and time courses (**a**) are shown. Key to **a** *black* 640 nm, *gray* 653 nm, *maroon* 666 nm, *brown* 678 nm, *red* 691 nm, *orange* 703 nm. Key to **b** *black* 0.3 s, *gray* 7.2 s, *maroon* 14.1 s, *brown* 21.0 s, *red* 27.9 s, *orange* 34.8 s. To this data matrix, we apply the SVD described by Eq. 1. The scree plot of the first 10 diagonal elements of ***S*** is shown in **e**. **c** and **d** display the first three *lsv* and *rsv* in *black*, *red* and *gray*, respectively

The original matrix Ψ can be satisfactorily reconstructed by means of the most significant singular vectors. A matrix is of rank *n* if *n* singular values differ significantly from the singular values that represent the noise. The rank of the data matrix is determined after visual inspection of the logarithmic plot of the diagonal elements of ***S*** (also called scree plot, e.g., Fig. 1e) and of the singular vectors (*e.g.*, Figure 1c, d). When the scree plot indicates that the data matrix is of rank 2, a matrix *A*

2 $$A = \left( {\begin{array}{*{20}c} 1 & {a_{12} } \\ {a_{21} } & {a_{21} \cdot a_{22} } \\ \end{array} } \right)$$

is introduced in Eq. 1 to write the decomposition of the data matrix as

3 $$\varPsi = U \cdot S \cdot (A^{ - 1} \cdot A) \cdot V^{T}$$

The aim is to find a biophysically meaningful solution by tuning the coefficients *a*_*12*_, *a*_*21*_, and *a*_*22*_. We note in Eq. 3 that **A** operates on the *rsv*, whereas **A** ^**−1**^ operates on $U \cdot S$, i.e., the *lsv* scaled by ***S***. The SAS are obtained after this transformation of the *rsv.* Explicitly, they fulfill

4 $$\left( {\begin{array}{*{20}c} {{\text{SAS}}_{1\lambda } } \\ {{\text{SAS}}_{2\lambda } } \\ \end{array} } \right) = AV^{T} = \left( {\begin{array}{*{20}c} 1 & {a_{12} } \\ {a_{21} } & {a_{21} \cdot a_{22} } \\ \end{array} } \right) \cdot \left( {\begin{array}{*{20}c} {v_{1\lambda } } \\ {v_{2\lambda } } \\ \end{array} } \right)$$

where the second index *λ* indicates that *v*_*1λ*_ and *v*_*2λ*_ are row vectors of length *n* (wavelengths). Expansion of Eq. 4 leads to two equations that can be written as follows:

5 $$\begin{aligned} &{\text{SAS}}_{1\lambda } = v_{1\lambda } + a_{12} \cdot v_{2\lambda } \hfill \\ &{\text{SAS}}_{2\lambda } = a_{21} (v_{1\lambda } + a_{22} \cdot v_{2\lambda } ) \hfill \\ \end{aligned},$$

where the respective coefficients acquire the following meaning: *a*_*12*_ is the *spectral shape factor* of SAS_1λ_, *a*_*22*_ is the *spectral shape factor* of SAS_2λ_, and *a*_*21*_ is a *scaling factor* that tunes the relative amplitudes of SAS_1λ_ and SAS_2λ_. Using the inverse of *A*,

6 $$A^{ - 1} = \frac{1}{{a_{21} (a_{22} - a_{!2} )}} \cdot \left( {\begin{array}{*{20}c} {a_{21} \cdot a_{22} } & { - a_{12} } \\ { - a_{21} } & 1 \\ \end{array} } \right)$$

we compute the matrix $C = U \cdot S \cdot A^{ - 1}$, that contains two column vectors for the concentrations *c*_*t1*_ and *c*_*t2*_ of length *m* (time points). Their explicit expression, according to Eq. 6, is:

7 $$\begin{aligned} c_{t1} = \frac{{a_{22} \cdot s_{1} \cdot u_{t1} - s_{2} \cdot u_{t2} }}{{a_{22} - a_{12} }} \hfill \\ c_{t2} = \frac{{s_{2} \cdot u_{t2} - a_{12} \cdot s_{1} \cdot u_{t1} }}{{a_{21} (a_{22} - a_{12} )}} \hfill \\ \end{aligned}$$

Hence, the final decomposition of a rank 2 data matrix Ψ can be expressed as the linear combination of these two concentrations and SAS:

8 $$\varPsi = c_{t1} SAS_{1\lambda } + c_{t2} SAS_{2\lambda }.$$

To judge whether the transformation yields a biophysically meaningful solution, the following four criteria are used: (i) non-negativity of the SAS, (ii) non-negativity of their difference (SAS_1λ_–SAS_2λ_), (iii) non-negativity of the concentrations, and (iv) constancy of their sum ($c_{t1} + c_{t2}$) during constant excitation. For a rank two matrix, criterion *iv* is equivalent to the assumption that an excitation can only be emitted either as SAS_1λ_ or SAS_2λ_. When the number of excitations created is constant, this means that the sum of concentrations is constant.

For richer data featuring a matrix of rank 3 (or higher), additional criteria would be needed to determine eight (or more) coefficients *a*_*ij*_.

## Results and discussion

Our spectra-decomposing method and analysis protocol were applied to two well-defined systems under different conditions. The first system studied (“OCP-related PB fluorescence quenching in vitro” section) is in vitro quenching of PB fluorescence by pre-converted OCP^r^, where only two states were expected (quenched and unquenched PB). The second system is the spectral transition during a single saturation pulse of white light with intact WT *Synechocystis* cells in vivo (“Closing of reaction centers during fluorescence induction by saturating light in vivo” section). These two systems are discussed in the following two sections. Each section consists of (i) description of the light protocol, (ii) description of the results, and (iii) conclusions. Based on this, the method is applied to more complex datasets of intact living cells acquired during orange light irradiation (“In vivo fluorescence induction with orange light in wild-type and PSI-, and PSII-deficient mutants of *Synechocystis*” section).

### OCP-related PB fluorescence quenching in vitro

The OCP–PB interaction has been reconstituted and characterized in vitro (Gwizdala et al. 2011; Jallet et al. 2012; Wilson et al. 2012). In particular, the effects on the kinetics of fluorescence quenching as a function of OCP/PB ratio have been investigated using a PAM fluorometer and we present here one of the protocols with the aim of validating our method (see the “Time-resolved fluorescence spectra at room temperature” section). What we expect to see is: evidence for PB in either an unquenched (before OCP^r^ is added) or a quenched state (once OCP^r^ binds to the PB core) (Gwizdala et al. 2011).

First, a selected visualization of the acquired data matrix is depicted in Fig. 1. Time-dependent fluorescence levels at specific wavelengths (Fig. 1a) and selected spectra at specific time instants (Fig. 1b) are shown. From these data, we can qualitatively conclude that the PB fluorescence is reduced upon binding of OCP^r^.

Figure 1c–e shows the results of SVD (see Eq. 1) of the data matrix. Panel *e* is the scree plot from which we conclude that the data matrix is of rank 2. In other words: only the first two *lsv* and *rsv* contribute with information distinguishable from the noise. Panels c and d illustrate this clearly: the first three *lsv,* ***u***_***t1***_ ***, u***_***t2***_, and ***u***_***t3***_ and the first three *rsv*, ***v***_***1λ***_, ***v***_***2λ***_, and ***v***_***3λ***_ are displayed in black, red, and gray, respectively. Clearly, the third components (***u***_***t3***_ and ***v***_***3λ***_, in gray) are not distinguishable from the noise.

**Fig. 2:**
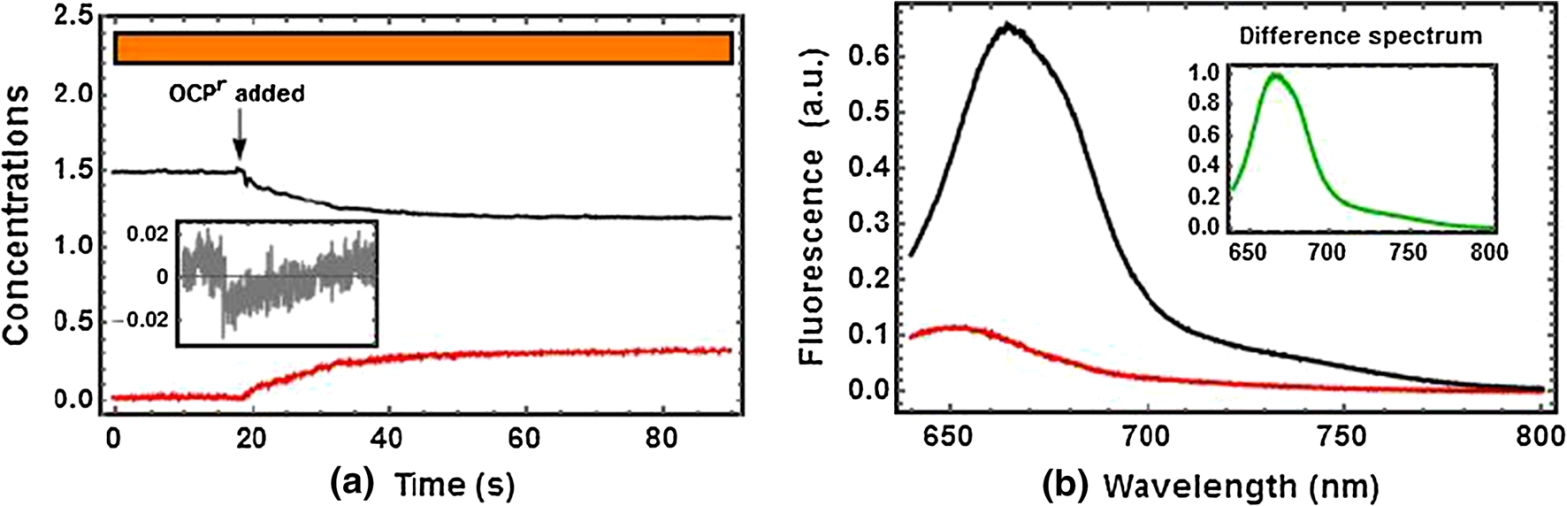
Decomposition of the time-resolved fluorescence spectrum of isolated PB during an OCP-induced quenching experiment. **a** Concentration profiles of the two components *c*_*1*_ (*black*) and *c*_*2*_ (*red*) according to Eq. 7. The *colored bar* on *top* illustrates the light regime: 300 µmol photons m^−2^ s^−1^ of 590 nm light; *Inset* The sum of concentrations remains fairly constant with 2 % of maximal variation from the average. **b** Species-associated spectra SAS_1,PBis_ (*black*) and SAS_2,PBis_(*red*). Transformation coefficients were set to *a*_*12*_ = −0.40, *a*_*21*_ = 6.35, and *a*_*22*_ = −0.01. *Inset* Difference spectrum SAS_1,PBis_–SAS_2,PBis_ normalized to its maximum (665 nm)

As a next step, a set of transformation coefficients (see Eq. 4) is determined to find the two characteristic SAS_1λ_ and SAS_2λ_ (SAS_1_ and SAS_2_ hereafter) and their concentrations c_t1_ and c_t2_ (c_1_ and c_2_ hereafter). The result is shown in Fig. 2. The concentration profiles are shown in Fig. 2a with an orange bar indicating continuous illumination with orange light. The inset shows the percent deviation of the sum of concentrations from the average. Figure 2b shows the SAS, that we call SAS_1,PBis_ and SAS_2,PBis_ hereafter (is = isolated). The difference between SAS_1,PBis_ (black) and SAS_2,PBis_ (red) is shown in the inset in green. The criteria to accept this result have been a) the non-negativity of both concentration profiles c_1_ and c_2_, b) a near-zero c_2_ before addition of OCP^r^, c) a sum of concentrations that deviates only 2 % from the average (inset), and d) non-negative SAS. OCP^r^ has been added at *t* ≈ 18 s. Before this time point, only SAS_1,PBis_ contributes to the signal. Upon addition of OCP^r^ and formation of the quenching complex PB-OCP^r^, an increase of c_2_ (corresponding to SAS_2,PBis_) at the expense of a decrease in c_1_ is observed and confirms reported kinetics (Gwizdala et al. 2011). We therefore conclude that SAS_1,PBis_ is the unquenched, while SAS_2,PBis_ is the quenched form of the isolated PB. Both are in a good agreement with emission spectra of (un)quenched PB reported in the past (Tian et al. 2012; Jallet et al. 2012; Gwizdala et al. 2011).

### Closing of reaction centers during fluorescence induction by saturating light in vivo

It is well known that a photosynthetic organism, when illuminated, displays a polyphasic increase in fluorescence (Kautsky and Hirsch 1931). After numerous reviews on the Kautsky effect, it has become common to speak about the so-called O-J-I-P steps of fluorescence induction (Lazár and Jablonský 2009; Stirbet et al. 2014; Govindjee 1995; Schansker et al. 2006; Stirbet and Govindjee 2011, 2012). Furthermore, slower transient characteristics (S-M-T) have also been observed and monitored (Kodru et al. 2015; Kaňa et al. 2012; Papageorgiou et al. 2007; Kaňa et al. 2009; Papageorgiou and Govindjee 2011). Our goal is to show how our analysis resolves, if any, contributions of different species during the OJIP regime, i.e., within the first second of illumination.

**Fig. 3:**
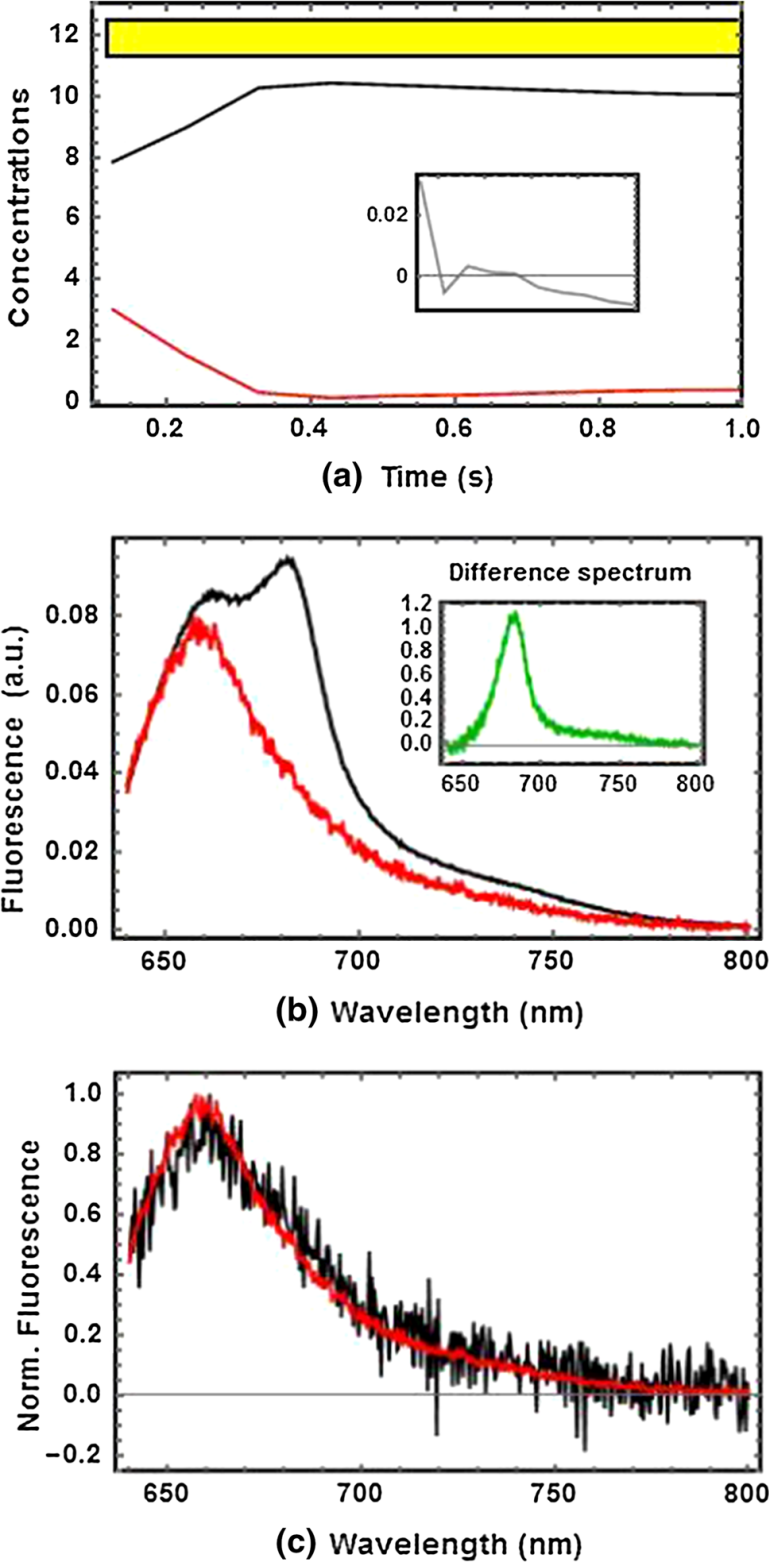
Decomposition of the time-resolved fluorescence spectrum from whole cells of wild-type *Synechocystis* during a saturation flash of white light (1300 µmol photons m^−2^ s^−1^) illustrated by the *yellow bar*. **a** Concentration profiles of the two components *c*_*1*_ (*black*) and *c*_*2*_ (*red*). *Inset* The sum of concentrations remains fairly constant within ≈1 % of maximal variation from the average. **b** Species-associated spectra SAS_1,sat_ (*black*) and SAS_2,sat_ (*red*) after transformation coefficients were set to *a*_*12*_ = −0.014; *a*_*21*_ = 0.64; *a*_*22*_ = 0.38. *Inset* Difference spectrum SAS_1,sat_–SAS_2,sat_ normalized to its maximum (682 nm) **c** *For comparison* normalized SAS_2,sat_ from panel **b** (*red*) and SAS from an independent, very low-intensity experiment on whole cells of *Synechocystis* (*black*)

The action of a single saturation pulse on dark-adapted *Synechocystis* cells has been investigated: a high-intensity orange pulse is applied during 1 s, while the fluorescence response is integrated every 100 ms (see “Time-resolved fluorescence spectra at room temperature” section). We expect to observe PSII activity only given that PSI not only does not display any closure dynamics, as PSII does, but it also contributes very little to the signal. The SVD is shown in Figure S2. The scree plot (Figure S2c) shows two singular values distinct from the noise. After multiplication with the *A* matrix, SAS_1,sat_ (black) and SAS_2,sat_ (red) as well as their time evolution are obtained (Fig. 3b). We note that, during the first 300 ms, *c*_*2*_ steadily decreases until it reaches a zero concentration (Fig. 3a). Concomitantly, *c*_*1*_ increases reaching a plateau for *t* > 0.3 s. The typical inflection points of the OJIP-curve cannot be assessed due to the integration time of 100 ms. Note, however, that full closure of the RCs is only achieved after ≈300 ms which lies within the expected time scale (Papageorgiou et al. 2007; Krause and Weis 1991; Neubauer and Schreiber 1987; Schreiber and Neubauer 1987). The difference spectrum clearly points at Chl *a* fluorescence, which could be explained by closure of PSII RCs. Therefore, we conclude that SAS_2,sat_ is a PB-PSII complex with *open* PSII RCs (PB-PSII_open_), while SAS_1,sat_ is a PB-PSII complex with *closed* PSII RCs (PB-PSII_closed_). We support this interpretation with an independent measurement carried out with very low intensity and very long integration time (1 µmol of photonsˑm^−2^ s^−1^; 10 s). The data matrix obtained under these illumination conditions was of rank 1 and the signal is expected to originate predominantly from PB-PSII complexes with *open* PSII RCs. Note that the black curve in Fig. 3c agrees well with SAS_2,sat_.

Summarizing, with the help of isolated systems the SAS of the (un)quenched isolated phycobilisome antenna could be estimated, and, by applying a single saturation pulse on intact WT *Synechocystis* cells, a PB-PSII complex in the open state could be distinguished from one in the closed state.

### In vivo fluorescence induction with orange light in wild-type and PSI-, and PSII-deficient mutants of *Synechocystis*

After having analyzed data obtained with simple protocols, the method was applied to analyze more complex experiments performed on whole cells of *Synechocystis*: these are continuously exposed to orange light of 300 µmol of photonsˑm^−2^ s^−1^ throughout the measurement and to 1500 µmol of photonsˑm^−2^ s^−1^ of the same light during the saturation flashes (clearly distinguishable peaks in the figures described below).

**Fig. 4:**
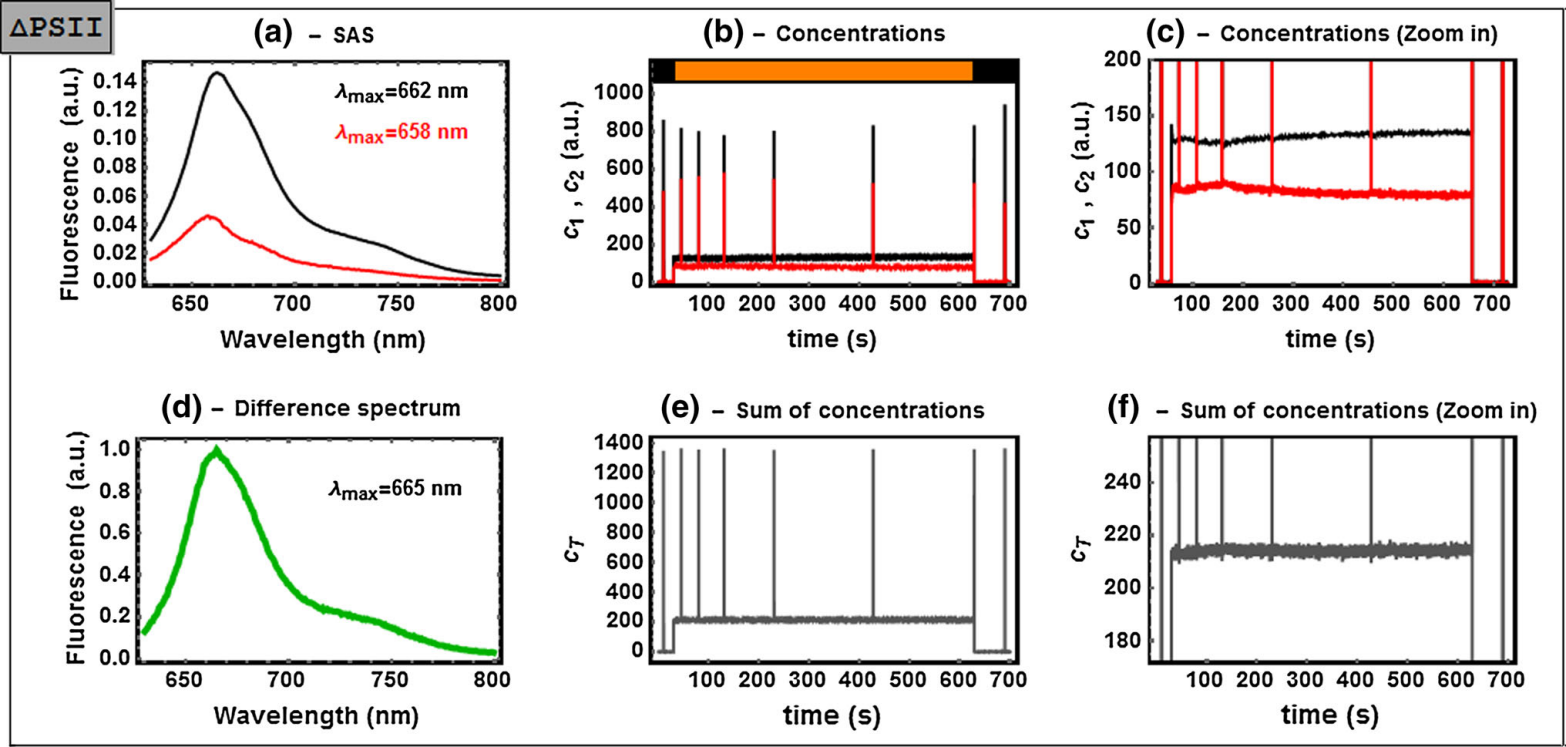
Analysis of the time-resolved fluorescence spectra of non-treated ΔPSII mutant cells. **a** Estimated SAS_1,ΔPSII_ (*black*) and SAS_2,ΔPSII_ (*red*). **b** the concentration profiles *c*_*1*_ (*black*) and *c*_*2*_ (*red*), the spikes are due to the saturation flashes; the illumination protocol is represented by the *colored bars* on *top* **c** zoom into a region of **b**, **d** the normalized difference spectrum between *black* and *red* SAS; **e** sum of the concentrations **c**_total_; **f** zoom into a region of **e**. *Transformation coefficients* a_12_ = 0.026, a_21_ = 0.3 and a_22_ = −0.16 (see the detailed explanation in the main text). The SVD and full analysis of this dataset are shown in Figs. S3 and S6, respectively

The data matrices are of rank 2 (see Figures S3–5). The analysis of the *Synechocystis* ΔPSII mutant yields the results as shown in Fig. 4. We associate SAS_1,ΔPSII_ with PB emission and SAS_2,ΔPSII_ with a quenched form of the PB. To resolve its origin, more datasets are necessary, however, given (i) the lack of fully assembled PSII RCs in this particular mutant and supported by (ii) the high amount of PSI complexes typically present in cyanobacterial thylakoids (Melis 1989; Moal and Lagoutte 2012; Shevela et al. 2013), a direct transfer from PB to PSI could be the origin of the quenching. Moreover, there is no DCMU-related effect on the dynamics (panel c of Figure S6). This is a sensible observation: DCMU is expected to block transfer from Q_A_^−^ (the primary quinone electron acceptor of PSII) to the plastoquinone pool (PQ) by binding to the Q_B_ site of the D1 protein (Krause and Weis 1991). Indeed, in a system where fully assembled PSII is absent, the use of DCMU should trigger no response from the PSII complex. It should be noted that the PSII mutant contains small amounts of PSII-core complexes [less than 10 % in comparison to WT; see Komenda et al. (2004)]. The influence of such remainders, however, is negligible.

**Fig. 5:**
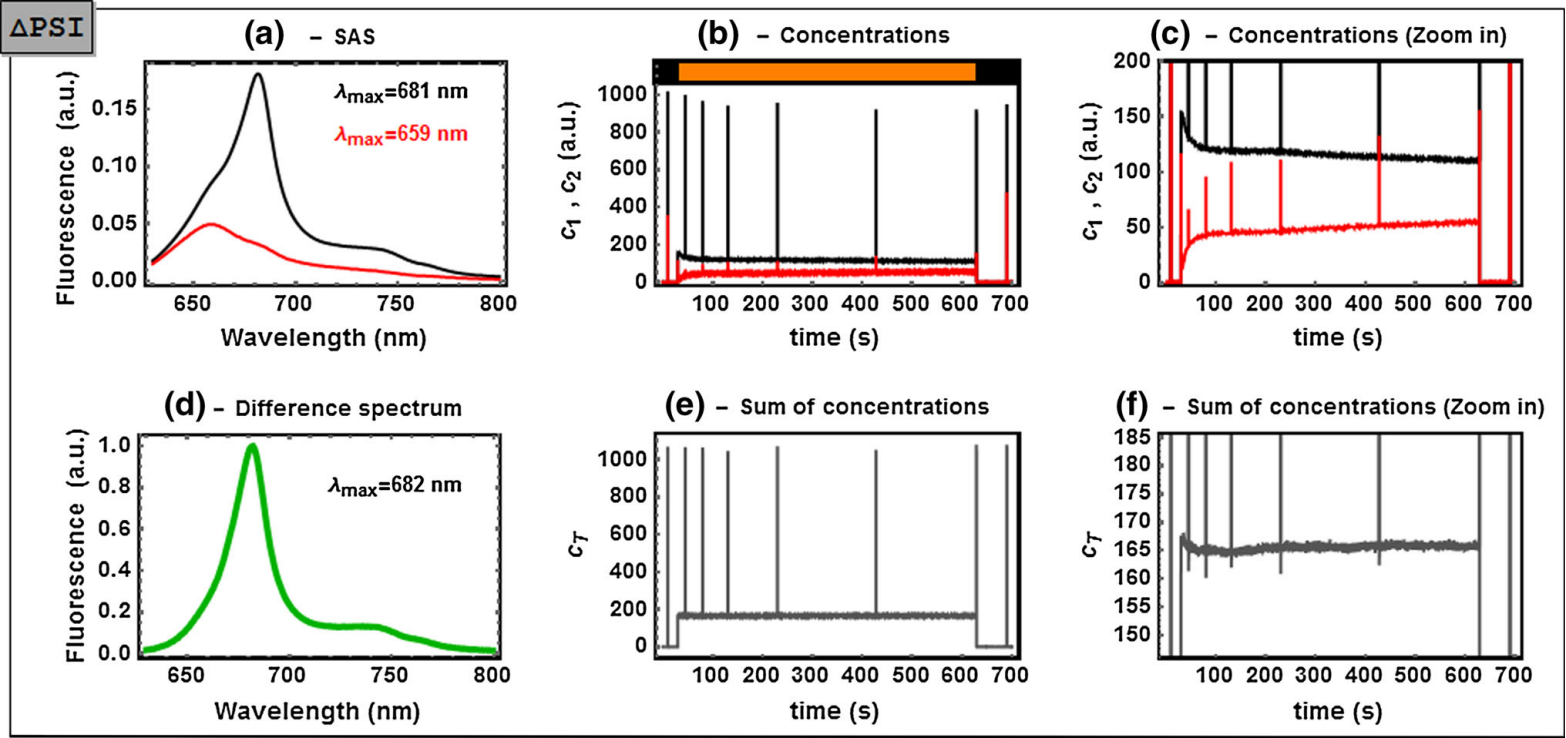
Analysis of the time-resolved fluorescence spectrum of non-treated ΔPSI mutant cells. **a** Estimated SAS_1,ΔPSI_ (*black*) and SAS_2,ΔPSI_ (*red*); **b** the concentration profiles *c*_*1*_ (*black*) and *c*_*2*_ (*red*); **c** zoom into a region of **b**, **d** the normalized difference spectrum between *black* and *red*; **e** sum of the concentrations **c**_total_; **f** zoom into a region of **e**. Transformation coefficients: a_12_ = 0.055, a_21_ = 0.32 and a_22_ = −0.45 (see detailed explanation in the main text). The SVD and full analysis of this dataset are shown in Figs. S4 and S7, respectively

The results obtained for the ΔPSI mutant are summarized in Figs. 5, S4 and S7. The SAS_1,ΔPSI_ (black) spectrum strongly differs from SAS_1,ΔPSII_ due to the presence of PSII RCs. In analogy to our interpretation of SAS_1,sat_, we interpret SAS_1,ΔPSI_ as PB-PSII_closed_. The SAS_2,ΔPSI_ spectrum has a relative amplitude that strongly suggests a quenched PB. The highest *c*_*2*_ values were reached via the saturation flashes during darkness (see first and last pulses in Fig. 5b). This contrasts with its rather small amplitude during the initial phase of orange light; it increased only slowly on light, reaching a steady level within ≈100 s (see zoom panel in Fig. 5c). This would have to involve an unknown, and rather untypical, quencher X that competes with energy transfer to PSII RCs in darkness (e.g., non-activated centers) and that is slowly activated by light on prolonged periods of irradiation. Interestingly, the concentration of SAS_2,ΔPSI_ remained maximal even during the light period in the presence of DCMU to close PSII (cf. Fig. S7c). One possibility for this rather untypical quencher may be a high light-inducible polypeptide (HliP), since (i) they presumably act as photoprotective players (He et al. 2001; Daddy et al. 2015; Komenda and Sobotka 2016) and (ii) they are overexpressed under various stressed conditions, including PSI deletion (see review by Komenda and Sobotka (2016) and references therein). Recently, quenching of Chl *a* Q_y_ by means of direct energy transfer to the S_1_ state of β-carotene in an HliP has been shown by Staleva et al. (2015). It suggests, indeed, involvement of an HliP protein in the quenching in the ΔPSI mutant.

**Fig. 6:**
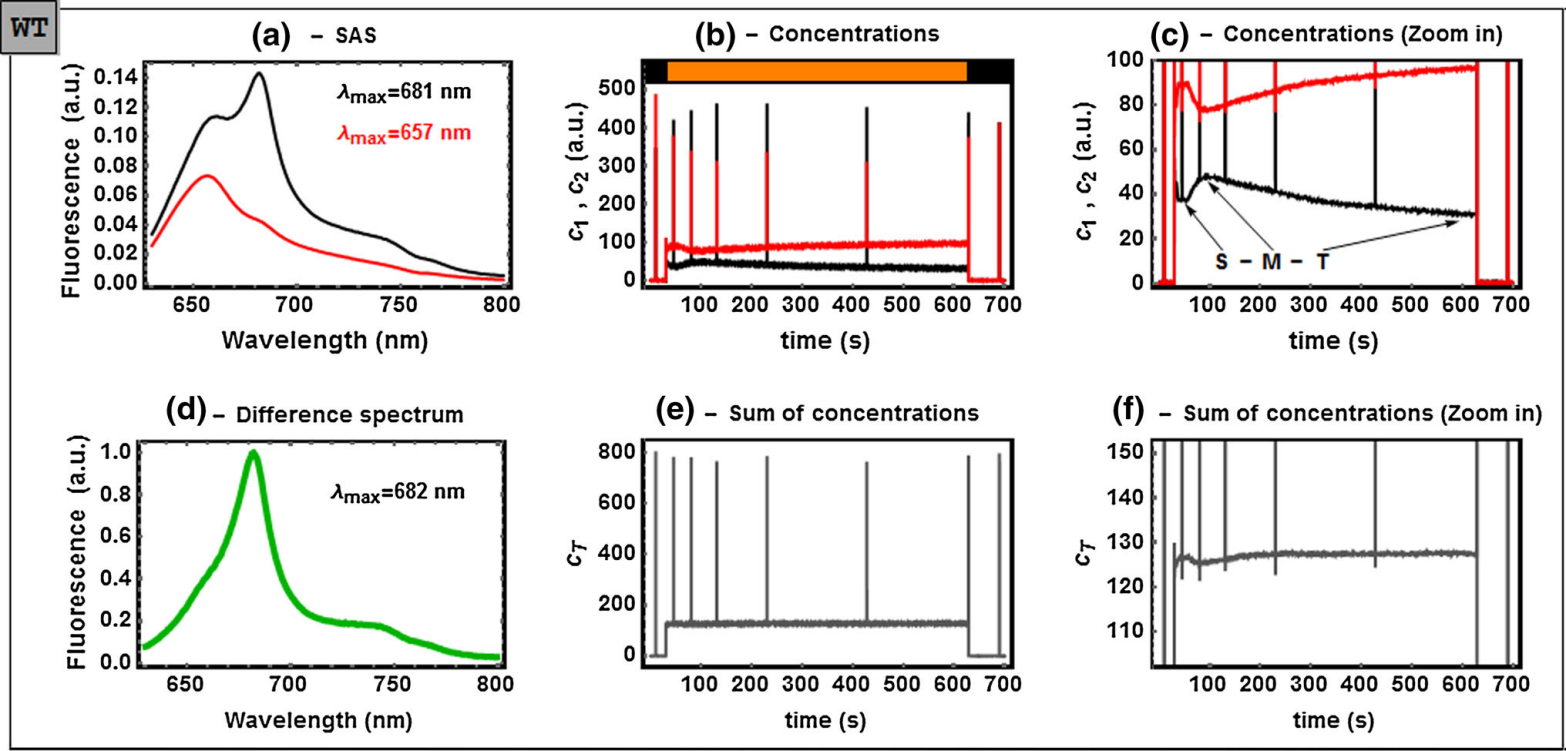
Analysis of the time-resolved fluorescence spectrum of non-treated WT cells. **a** Estimated SAS_1,WT_ (*black*) and SAS_2,WT_ (*red*); **b** the concentration profiles *c*_*1*_ (*black*) and *c*_*2*_ (*red*); **c** zoom into a region of **b,** **d** the normalized difference spectrum between *black* and *red*; **e** sum of the concentrations **c**_total_; **f** zoom into a region of **e**. Transformation coefficients: a_12_ = 0.16, a_21_ = 0.51 and a_22_ = −0.15 (see detailed explanation in the main text). The SVD and full analysis of this dataset are shown in Figs. S5 and S8, respectively

**Fig. 7:**
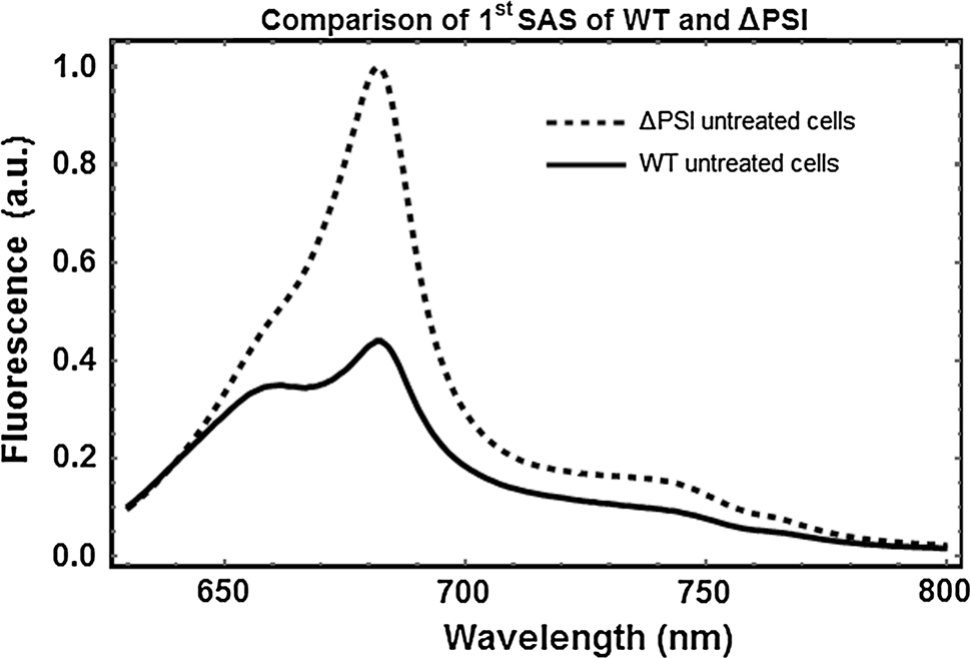
Overlay plot of the SAS_1,WT_ and SAS_1,ΔPSI_. The scaling is such that the respective blue edges (associated with PC emission) overlap

The results obtained for the WT cells are summarized in Fig. 6, S5 and S8. In this case, *c*_*1*_ exhibits the slow S to M (to T) transition (Fig. 6c) related to the state 2 to 1 transition (Kaňa et al. 2012). As for the nature of the SAS, we associate SAS_1,WT_ with PB-PSII_closed_. The SAS_2,WT_ features a quenched, hybrid form: it appears quenched relative to SAS_1,WT_ and its shape does not entirely match those of SAS_2,ΔPSII_ or SAS_2,ΔPSI_. Also, we note that the ratio F650/F680 in SAS_1,WT_ remarkably differs from that of SAS_1,ΔPSI_. This is demonstrated in Fig. 7, where SAS_1,WT_ has been scaled such that the respective regions below 650 nm (associated with PC emission from the PB antenna) overlap. In both cases, SAS_1,ΔPSI_ and SAS_1,WT_, have been assigned to PB-PSII_closed_. How to explain the striking difference uncovered by Fig. 7 then? We put forward the following hypothesis: SAS_1,ΔPSI_ would be interpreted as a *pure* species PB-PSII_closed_, since the system consists of PB and PSII only. When PSI is added to the complex, as it is in WT, excitation energy can be transferred to PSI, where it is efficiently quenched. As a result, the 680 nm emission of the PSII with closed RCs is reduced, yielding SAS_1,WT_. We call this *mildly* quenched by PSI. Before the state 2 to state 1 transition takes place, *c*_*2*_ reaches a maximum (minimum of *c*_*1*_ indicated by “S” in Fig. 6c) due to an increased excitation energy transfer rate to PSI. Therefore, in our interpretation, SAS_2,WT_, where there is little 680 nm emission left, is the signature of a *strongly* quenched complex. This interpretation is in agreement with the previously reported PB-PSII-PSI megacomplex (Liu et al. 2013). Our current method is not able to discard alternative models for state transitions that involve, for instance, *spill*-*over*, i.e., the direct energy transfer from PSII to PSI (McConnell et al. 2002; Kirilovsky et al. 2014). We argue, however, that small nano-scale “switching”-like re-arrangements of these super complexes between a complex with a mildly/strongly coupled quencher could provide a fundamental clue on the mechanism of the state transition. These interpretations are also in line with the much faster increase/decrease in the SAS_1,WT_/SAS_2,WT_ component with DCMU (see panel C in Figure S8). State 2 to 1 transition acceleration with this inhibitor has been, indeed, reported by Kaňa et al. (2012).

**Fig. 8:**
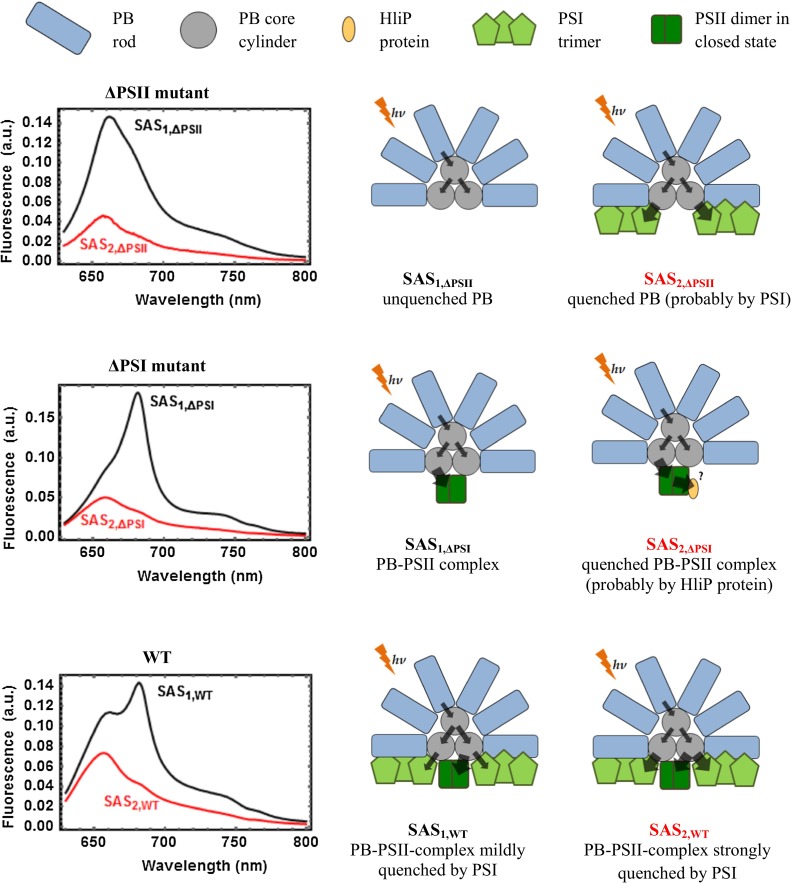
Proposed interpretation of the SAS obtained in the analysis of all samples. The inferred species shown are represented next to each pair of SAS. *Top* The ΔPSII mutant data feature unquenched PB and quenched PB via PSI. *Center* The ΔPSI mutant data feature a PB-PSII complex in closed state in an unquenched or a quenched state [probably by a high light-inducible protein, cf. Komenda and Sobotka (2016)]. *Bottom* In the WT, the presence of PSI and PSII leads to formation of a PB-PSII-PSI megacomplex (Liu et al. 2013) that can be either *mildly* or *strongly* quenched. *Arrows* indicate the flow of energy from the PB rods to the PB core cylinders, and from there to PS II or to PS I. The width of an arrow indicates the rate of energy transfer [estimated after Tian et al. (2011)]

Our interpretations are summarized in Fig. 8. Phycobilisomes, PSII dimers, and PSI trimers are represented in a cartoon-like manner and not to scale. Energy transfer from the PB rods to the PB core, and from the PB core to the PSI and PSII units is illustrated by black arrows, whose size is an indication of its efficiency. For instance, based on quantitative analysis performed by Tian et al. (2011), we represent energy transfer times (*e.g.*, from the PB rods to the PB core) on the order of 100 ps with a slim arrow, whereas faster energy transfer times (from the terminal emitters in the PB core to PSII) are represented by a thick arrow. Note that back transfer rates (from the PB core to the PB rods and from PSII to the terminal emitters in the PB core) are an order of magnitude smaller, and have been omitted in the cartoons. Thus, in the bottom row, the difference between SAS_1,WT_ and SAS_2,WT_ is visualized as an increase in the transfer rate from the PB core to PS I. This hypothesis remains to be tested.

## Conclusions

We have presented in this paper a method to analyze time-dependent spectrally resolved fluorometry data, acquired using a multi-source fluorometer set-up. We have shown that in all cases presented here, the data can be decomposed into two SAS and their time evolution. By first applying the method to datasets acquired by means of simple protocols (isolated PB during OCP-induced quenching, WT cells during a saturation flash), the SAS of isolated un/quenched PB and PB-PSII complexes in either open or closed state have been determined.

This method was then applied to WT and selected mutants of *Synechocystis* during different light conditions. In these cases, too, data matrices were well approximated by rank two matrices and for each pair of derived SAS, hypotheses about their molecular origin based upon the existing literature and sensible assumptions have been put forward. These interpretations are summarized in Fig. 8. The most striking finding concerns the difference between the respective SAS_1_ of WT and the ΔPSI mutant (see Figs. 7, 8) which we interpret as evidence for *mild* quenching by PSI in the WT PB-PSII-PSI megacomplex. While in state 2, the energy transfer to the PSI moiety of the megacomplex accelerates resulting in the quenched second SAS of WT (see Figs. 6 and 8). The wealth of information hidden in the SAS and the time traces of their concentrations presented here calls for the development of models at the molecular level to solidify their interpretation.

## Electronic supplementary material

Below is the link to the electronic supplementary material.

Supplementary material 1 (DOCX 508 kb)

## References

[CR1] Campbell D, Hurry V, Clarke AK, Gustafsson P, Öquist G (1998). Chlorophyll fluorescence analysis of cyanobacterial photosynthesis and acclimation. Microbiol Mol Biol Rev.

[CR2] Cho F, Govindjee (1970). Low-temperature (4–77 °K) spectroscopy of anacystis; temperature dependence of energy transfer efficiency. Biochim Biophys Acta.

[CR3] Chukhutsina V, Bersanini L, Aro E-M, van Amerongen H (2015). Cyanobacterial Light-harvesting phycobilisomes uncouple from photosystem i during dark-to-light transitions. Sci Rep.

[CR4] Daddy S, Zhan J, Jantaro S, He C, He Q, Wang Q (2015). A novel high light-inducible carotenoid-binding protein complex in the thylakoid membranes of *Synechocystis* PCC 6803. Sci Rep.

[CR5] Demmig-Adams B, Garab G, Adams W, Govindjee (2014). Non-photochemical quenching and energy dissipation in plants, algae and cyanobacteria. Advances in photosynthesis and respiration including bioenergy and related processes.

[CR6] Glazer AN, Bryant DA (1975). Allophycocyanin B (λmax 671, 618 nm). Arch Microbiol.

[CR7] Golub GH, Van Loan CF (1996). Matrix computations.

[CR8] Govindjee (1995). Sixty-three years since Kautsky: chlorophyll a fluorescence. Aust J Plant Physiol.

[CR9] Govindjee, Shevela D (2011). Adventures with cyanobacteria: a personal perspective. Front Plant Sci.

[CR10] Gwizdala M, Wilson A, Kirilovsky D (2011). In vitro reconstitution of the cyanobacterial photoprotective mechanism mediated by the orange carotenoid protein in *Synechocystis* PCC 6803. Plant Cell.

[CR11] He Q, Dolganov N, Björkman O, Grossman AR (2001). The high light-inducible polypeptides in *Synechocystis* PCC6803: expression and function in high light. J Biol Chem.

[CR12] Jallet D, Gwizdala M, Kirilovsky D (2012). ApcD, ApcF and ApcE are not required for the orange carotenoid protein related phycobilisome fluorescence quenching in the cyanobacterium *Synechocystis* PCC 6803. Biochim Biophys Acta.

[CR13] Joshua S, Mullineaux CW (2004). Phycobilisome diffusion is required for light-state transitions in cyanobacteria. Plant Physiol.

[CR14] Kaňa R, Prášil O, Komárek O, Papageorgiou GC, Govindjee (2009). Spectral characteristic of fluorescence induction in a model cyanobacterium, *Synechococcus* sp. (PCC 7942). Biochim Biophys Acta.

[CR15] Kaňa R, Kotabová E, Komárek O, Šedivá B, Papageorgiou GC, Govindjee, Prášil O (2012). The slow S to M fluorescence rise in cyanobacteria is due to a state 2 to state 1 transition. Biochim Biophys Acta.

[CR16] Kautsky H, Hirsch A (1931). Neue Versuche zur Kohlensäureassimilation. Naturwissenschaften.

[CR17] Kirilovsky D, Kaňa R, Prášil O, Demmig-Adams B, Garab G, Adams W, Govindjee (2014). Mechanisms modulating energy arriving at reaction centers in cyanobacteria. Non-photochemical quenching and energy dissipation in plants, algae and cyanobacteria. Advances in photosynthesis and respiration.

[CR18] Kodru S, Malavath T, Devadasu E, Nellaepalli S, Stirbet A, Subramanyam R, Govindjee (2015). The slow S to M rise of chlorophyll *a* fluorescence reflects transition from state 2 to state 1 in the green alga *Chlamydomonas reinhardtii*. Photosynth Res.

[CR19] Komenda J, Sobotka R (2016). Cyanobacterial high-light-inducible proteins—protectors of chlorophyll–protein synthesis and assembly. Biochim Biophys Acta.

[CR20] Komenda J, Reisinger V, Müller BC, Dobáková M, Granvogl B, Eichacker LA (2004). Accumulation of the D2 protein is a key regulatory step for assembly of the photosystem II reaction center complex in *Synechocystis* PCC 6803. J Biol Chem.

[CR21] Komura M, Itoh S (2009). Fluorescence measurement by a streak camera in a single-photon-counting mode. Photosynth Res.

[CR22] Krause GH, Weis E (1991). Chlorophyll fluorescence and photosynthesis: the basics. Annu Rev Plant Physiol Plant Mol.

[CR23] Lambrev PH, Nilkens M, Miloslavina Y, Jahns P, Holzwarth AR (2010). Kinetic and spectral resolution of multiple nonphotochemical quenching components in arabidopsis leaves. Plant Physiol.

[CR24] Lazár D, Jablonský J (2009). On the approaches applied in formulation of a kinetic model of photosystem II: different approaches lead to different simulations of the chlorophyll *a* fluorescence transients. J Theor Biol.

[CR25] Liu L-N (2015). Distribution and dynamics of electron transport complexes in cyanobacterial thylakoid membranes. Biochim Biophys Acta.

[CR26] Liu H, Zhang H, Niedzwiedzki DM, Prado M, He G, Gross ML, Blankenship RE (2013). Phycobilisomes supply excitations to both photosystems in a megacomplex in cyanobacteria. Science.

[CR27] McConnell MD, Koop R, Vasil’ev S, Bruce D (2002). Regulation of the distribution of chlorophyll and phycobilin-absorbed excitation energy in cyanobacteria. A structure-based model for the light state transition. Plant Physiol.

[CR28] Melis A (1989). Spectroscopic methods in photosynthesis: photosystem stoichiometry and chlorophyll antenna size. Philos Trans R Soc Lond B.

[CR29] Moal G (1817). Lagoutte B (2012) Photo-induced electron transfer from photosystem I to NADP+: characterization and tentative simulation of the in vivo environment. Biochim Biophys Acta.

[CR30] Mullineaux CW (2014). Electron transport and light-harvesting switches in cyanobacteria. Front Plant Sci.

[CR31] Mullineaux CW, Allen JF (1986). The state 2 transition in the cyanobacterium *Synechococcus* 6301 can be driven by respiratory electron flow into the plastoquinone pool. FEBS Lett.

[CR32] Mullineaux CW, Allen JF (1990). State 1-State 2 transitions in the cyanobacterium *Synechococcus* 6301 are controlled by the redox state of electron carriers between photosystems I and II. Photosynth Res.

[CR33] Mullineaux CW, Emlyn-Jones D (2005). State transitions: an example of acclimation to low-light stress. J Exp Bot.

[CR34] Nedbal L, Trtílek M, Červený J, Komárek O, Pakrasi HB (2008). A photobioreactor system for precision cultivation of photoautotrophic microorganisms and for high-content analysis of suspension dynamics. Biotechnol Bioeng.

[CR35] Neubauer C, Schreiber U (1987). The polyphasic rise of chlorophyll fluorescence upon onset of strong continuous illumination: I. Saturation characteristics and partial control by the photosystem II acceptor side. Zeitschrift für Naturforschung C.

[CR36] Nishiyama Y, Yamamoto H, Allakhverdiev SI, Inaba M, Yokota A, Murata N (2001). Oxidative stress inhibits the repair of photodamage to the photosynthetic machinery. EMBO J.

[CR37] Papageorgiou GC (1996). The photosynthesis of cyanobacteria (blue bacteria) from the perspective of signal analysis of chlorophyll *a* fluorescence. J Sci Ind Res.

[CR38] Papageorgiou GC, Govindjee (2011). Photosystem II fluorescence: slow changes—scaling from the past. J Photochem Photobiol, B.

[CR39] Papageorgiou GC, Tsimilli-Michael M, Stamatakis K (2007). The fast and slow kinetics of chlorophyll *a* fluorescence induction in plants, algae and cyanobacteria: a viewpoint. Photosynth Res.

[CR40] Schansker G, Tóth SZ, Strasser RJ (2006). Dark recovery of the Chl a fluorescence transient (OJIP) after light adaptation: the qT-component of non-photochemical quenching is related to an activated photosystem I acceptor side. Biochim Biophys Acta.

[CR41] Schreiber U, Neubauer C (1987). The polyphasic rise of chlorophyll fluorescence upon onset of strong continuous illumination: II. Partial control by the photosystem II donor side and possible ways of interpretation. Zeitschrift für Naturforschung C.

[CR42] Shen G, Boussiba S, Vermaas WFJ (1993). *Synechocystis* sp PCC 6803 strains lacking photosystem I and phycobilisome function. Plant Cell.

[CR43] Shevela D, Pishchalnikov RY, Eichacker LA, Govindjee, Srivastava AK, Rai AN, Neilan BA (2013). Oxygenic photosynthesis in cyanobacteria. Stress biology of cyanobacteria. Moleular mechanisms to cellular responses.

[CR44] Staleva H, Komenda J, Shukla MK, Šlouf V, Kaňa R, Polívka T, Sobotka R (2015). Mechanism of photoprotection in the cyanobacterial ancestor of plant antenna proteins. Nat Chem Biol.

[CR45] Stanier R, Kunisawa R, Mandel M, Cohen-Bazire G (1971). Purification and properties of unicellular blue-green algae (order *Chroococcales*). Bacteriol Rev.

[CR46] Steinbach G, Schubert F, Kaňa R (2015). Cryo-imaging of photosystems and phycobilisomes in *Anabaena sp. PCC 7120* cells. J Photochem Photobiol, B.

[CR47] Stirbet A, Govindjee (2011). On the relation between the Kautsky effect (chlorophyll a fluorescence induction) and photosystem II: basics and applications of the OJIP fluorescence transient. J Photochem Photobiol, B.

[CR48] Stirbet A, Govindjee (2012). Chlorophyll *a* fluorescence induction: a personal perspective of the thermal phase, the J-I-P rise. Photosynth Res.

[CR49] Stirbet A, Riznichenko GY, Rubin AB, Govindjee (2014). Modeling chlorophyll *a* fluorescence transient: relation to photosynthesis. Biochem (Mosc).

[CR50] Tian L, van Stokkum IHM, Koehorst RBM, Jongerius A, Kirilovsky D, van Amerongen H (2011). Site, rate, and mechanism of photoprotective quenching in cyanobacteria. J Am Chem Soc.

[CR51] Tian L, Gwizdala M, van Stokkum Ivo HM, Koehorst Rob BM, Kirilovsky D, van Amerongen H (2012). Picosecond kinetics of light harvesting and photoprotective quenching in wild-type and mutant phycobilisomes isolated from the cyanobacterium *Synechocystis* PCC 6803. Biophys J.

[CR52] van Alphen P, Hellingwerf KJ (2015). Sustained circadian rhythms in continuous light in *Synechocystis* sp. PCC6803 growing in a well-controlled photobioreactor. PLoS ONE.

[CR53] Vernotte C, Astier C, Olive J (1990). State 1-state 2 adaptation in the cyanobacteria *Synechocystis* PCC 6714 wild type and *Synechocystis* PCC 6803 wild type and phycocyanin-less mutant. Photosynth Res.

[CR54] Wilson A, Punginelli C, Gall A, Bonetti C, Alexandre M, Routaboul J-M, Kerfeld CA, van Grondelle R, Robert B, Kennis JTM, Kirilovsky D (2008). A photoactive carotenoid protein acting as light intensity sensor. Proc Natl Acad Sci USA.

[CR55] Wilson A, Gwizdala M, Mezzetti A, Alexandre M, Kerfeld CA, Kirilovsky D (2012). The essential role of the N-terminal domain of the orange carotenoid protein in cyanobacterial photoprotection: importance of a positive charge for phycobilisome binding. Plant Cell.

