# Supplementary material for: A method to decompose spectral changes in Synechocystis PCC 6803 during light-induced state transitions
Source: Photosynth Res. 2016 Mar 25;130(1):237–49. doi: 10.1007/s11120-016-0248-8 (PMC5054063; doi:10.1007/s11120-016-0248-8)
Supplement: Supplementary file 1 — Supplementary material 1 (DOCX 508 kb) [file 11120_2016_248_MOESM1_ESM.docx]

## Supplemental Information

This section contains (i) a graphical description of the multiple-LED set-up in Amsterdam, (ii) the SVD results of all experiments presented in the main text and (iii) the results obtained after analysis based upon our method for WT *Synechocystis* and the ΔPSI and ΔPSII mutants thereof both non-treated and in the presence of DCMU.

| **A**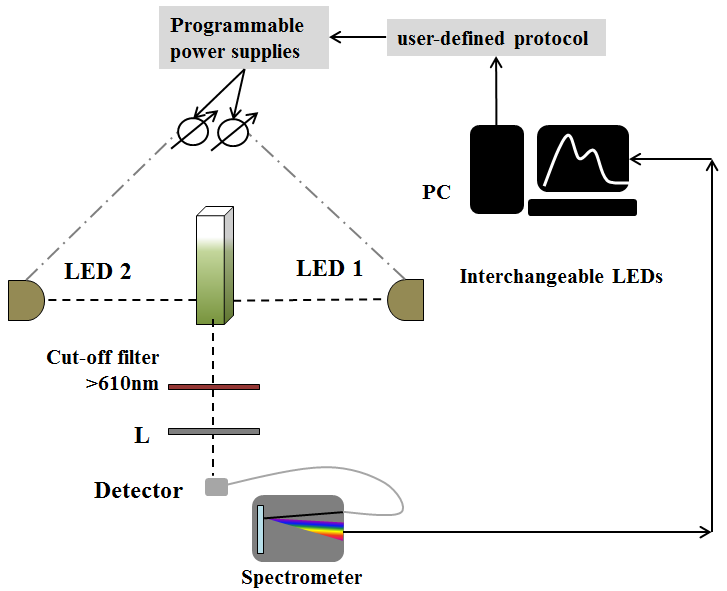 | **B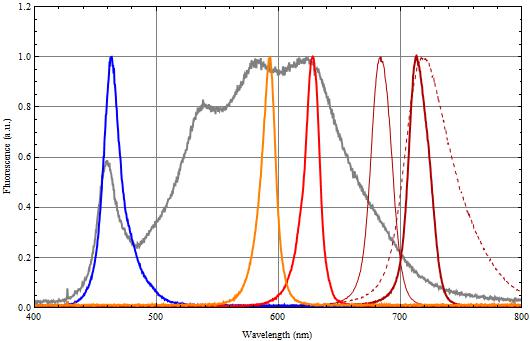** |
| --- | --- |

Figure S 1 A) Schematic representation of the multiple-LED set-up in the LaserLab, in Amsterdam, The Netherlands. B) Spectra of the LEDs available. Key: *Gray:* White LED; *Blue:* 460 nm; *Orange:* 590 nm; *Red:* 640 nm; *Dark red solid:* White LED with a 695 nm filter; *Dark red thick:* White LED with a 715 nm filter; *Dark red dashed:* White LED with a 725 nm filter

|  |
| --- |
| Figure S 2 SVD of the time resolved spectrum of the WT *Synechocystis* cells during a saturation flash. a) first two left singular vectors u_1_(black) and u_2_ (red). b) first two right singular vectors v_1_(black) and v_2_ (red). c) scree plot |

.

|  |
| --- |
| Figure S 3 SVD of the time resolved spectrum of the ΔPSII mutant cells. a) first two left singular vectors u_1_(black) and u_2_ (red). b) first two right singular vectors v_1_(black) and v_2_ (red). c) scree plot. |

Figure S 4 SVD of the time resolved spectrum of the ΔPSI mutant cells. a) first two left singular vectors u_1_(black) and u_2_ (red). b) first two right singular vectors v_1_(black) and v_2_ (red). c) scree plot.

Figure S 5 SVD of the time resolved spectrum of the WT cells. a) first two left singular vectors u_1_(black) and u_2_ (red). b) first two right singular vectors v_1_(black) and v_2_ (red). c) scree plot.

| 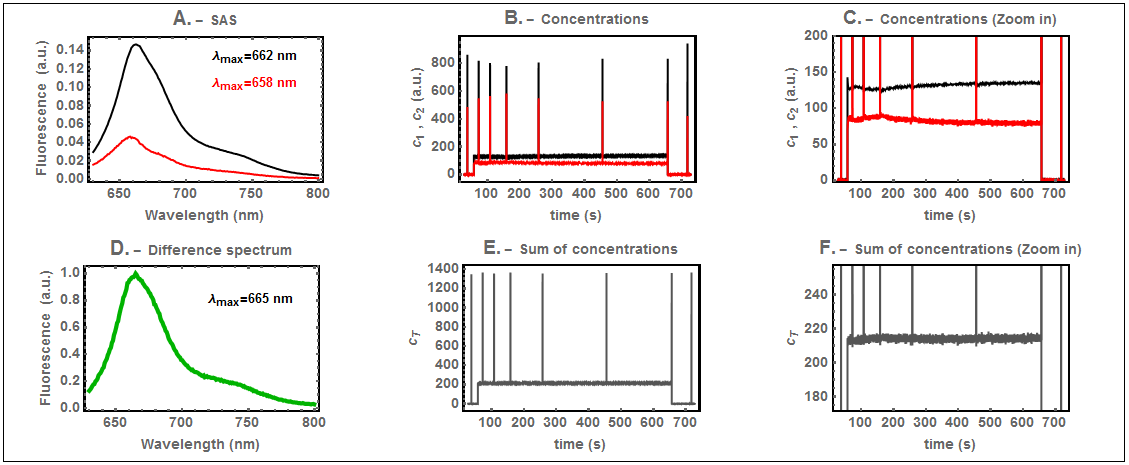  **ΔPSII**  **non-treated** |
| --- |
| 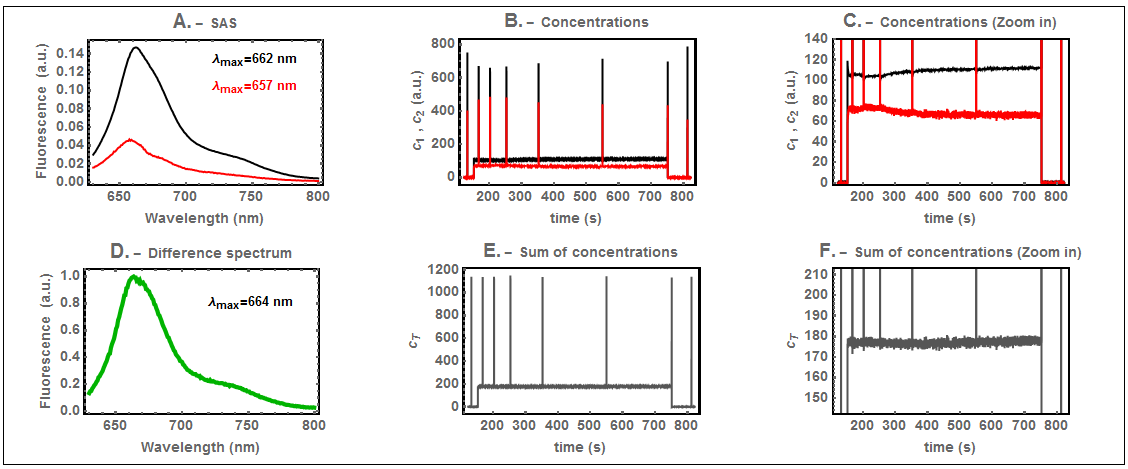  **ΔPSII**  **DCMU** |
| Figure S 6 Full analysis of the time resolved fluorescence spectrum of **ΔPSII mutant** cells: non-treated (above), DCMU-treated (below); **A:** Estimated SAS_1,ΔPSII_ (black) and SAS_2,ΔPSII_ (red). **B:** the concentration profiles *c_1_* (black) and *c_2_* (red), the spikes are due to the saturation flashes; **C:** zoom into a region of **B**; **D:** the normalized difference spectrum between black and red SAS; **E:** sum of the concentrations **c**_total_; **F:** zoom into a region of **E**. See the main text for a detailed explanation of the non-treated panels. Transformation coefficients: non-treated: a_12_= 0.026, a_21_= 0.3 and a_22_= – 0.16 ; DCMU: a_12_= 0.026, a_21_= 0.3 and a_22_= – 0.16 |
| 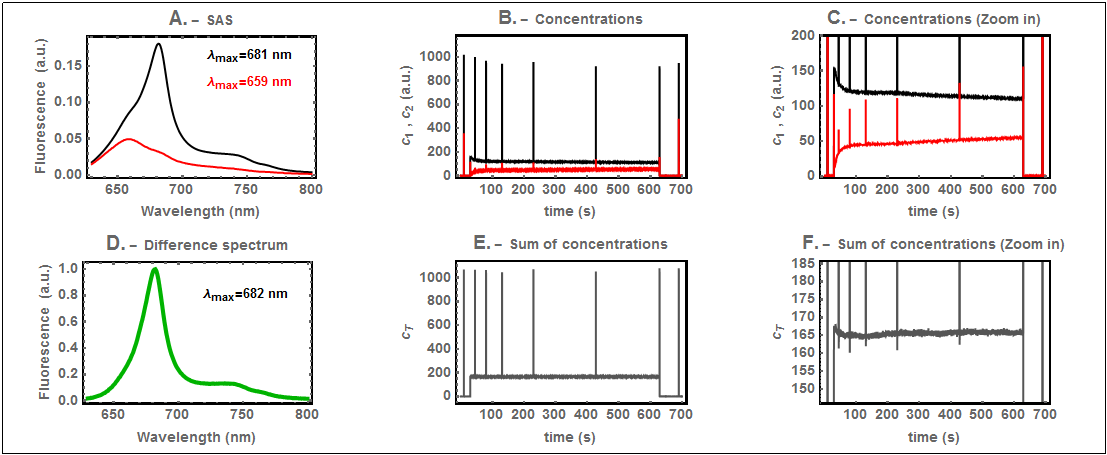  **ΔPSI**  **non-treated** |
| 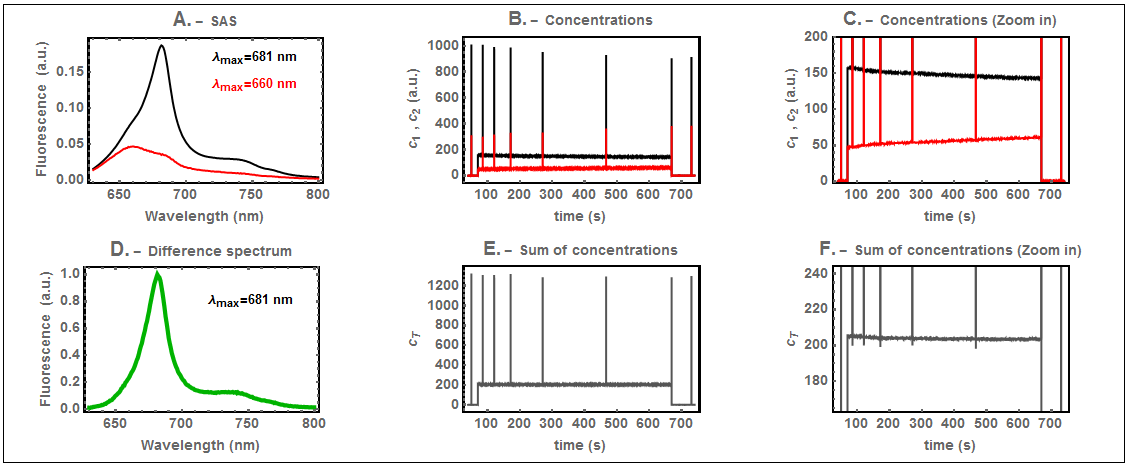  **ΔPSI**  **DCMU** |
| Figure S 7 Full analysis of the time resolved fluorescence spectrum of **ΔPSI mutant** cells: non-treated (above), DCMU-treated (below); **A:** Estimated SAS_1,ΔPSI_ (black) and SAS_2,ΔPSI_ (red); **B:** the concentration profiles *c_1_* (black) and *c_2_* (red); **C:** zoom into a region of **B**; **D:** the normalized difference spectrum between black and red; **E:** sum of the concentrations **c**_total_; **F:** zoom into a region of **E**. See the main text for a detailed explanation of the non-treated panels. Transformation coefficients: non-treated: a_12_= 0.055, a_21_= 0.32 and a_22_= – 0.45; DCMU: a_12_= 0.05, a_21_= 0.32 and a_22_= – 0.42 |

| 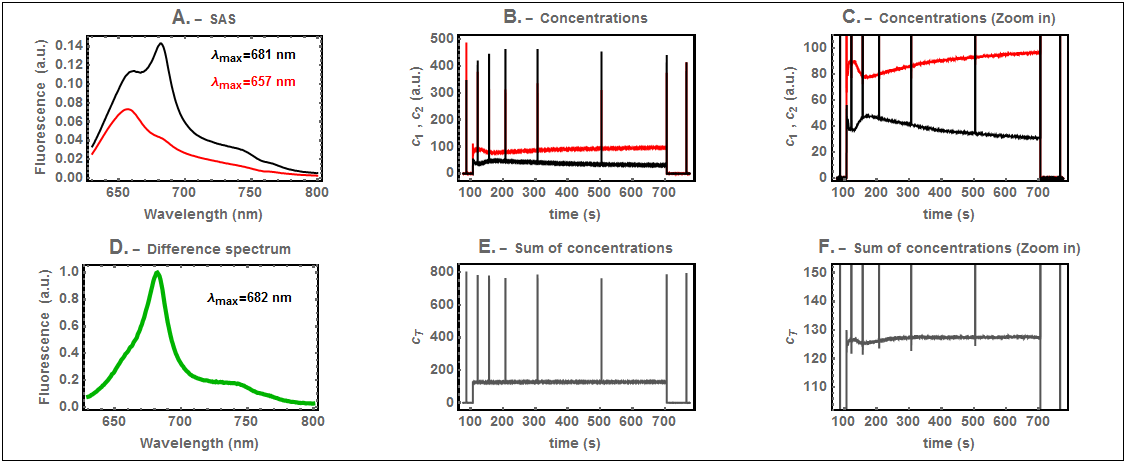  **WT**  **non-treated** |
| --- |
| 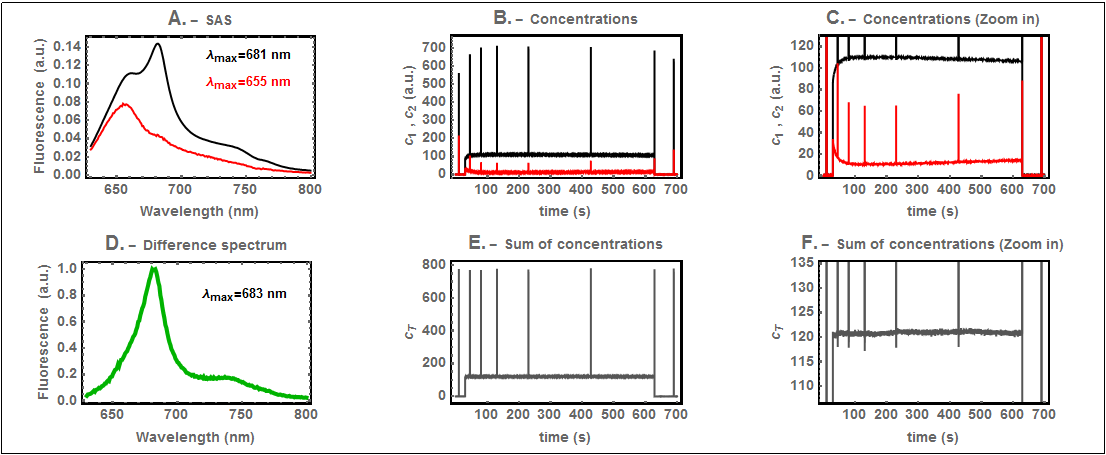  **WT**  **DCMU** |
| Figure S 8 Full analysis of the time resolved fluorescence spectrum of **WT** **cells**: non-treated (above), DCMU-treated (below); **A:** Estimated SAS_1,WT_ (black) and SAS_2,WT_ (red); **B:** the concentration profiles *c_1_* (black) and *c_2_* (red), where, *c_1_* has been pulled to the front for better visibility of the first/last pulses; **C:** zoom into a region of **B**; **D:** the normalized difference spectrum between black and red; **E:** sum of the concentrations **c**_total_; **F:** zoom into a region of **E**. See the main text for a detailed explanation of the non-treated panels. Transformation coefficients: non-treated: a_12_=  0.16, a_21_= 0.51 and a_22_= –0.15; DCMU: a_12_= – 0.02, a_21_= 0.51 and a_22_= 0.33 |
